# Transcriptome analysis of intraspecific competition in *Arabidopsis thaliana* reveals organ-specific signatures related to nutrient acquisition and general stress response pathways

**DOI:** 10.1186/1471-2229-12-227

**Published:** 2012-11-29

**Authors:** Frédéric G Masclaux, Friederike Bruessow, Fabian Schweizer, Caroline Gouhier-Darimont, Laurent Keller, Philippe Reymond

**Affiliations:** 1 Department of Plant Molecular Biology, University of Lausanne, Lausanne, 1015, Switzerland; 2 Present address: Max Planck Institute for Plant Breeding Research, Carl-von-Linne-Weg 10, Köln, 50829, Germany; 3 Department of Ecology and Evolution, University of Lausanne, Lausanne, 1015, Switzerland

**Keywords:** *Arabidopsis thaliana*, Gene expression, Intraspecific competition, Herbivory, Abiotic stress, Biotic stress

## Abstract

**Background:**

Plants are sessile and therefore have to perceive and adjust to changes in their environment. The presence of neighbours leads to a competitive situation where resources and space will be limited. Complex adaptive responses to such situation are poorly understood at the molecular level.

**Results:**

Using microarrays, we analysed whole-genome expression changes in *Arabidopsis thaliana* plants subjected to intraspecific competition. The leaf and root transcriptome was strongly altered by competition. Differentially expressed genes were enriched in genes involved in nutrient deficiency (mainly N, P, K), perception of light quality, and responses to abiotic and biotic stresses. Interestingly, performance of the generalist insect *Spodoptera littoralis* on densely grown plants was significantly reduced, suggesting that plants under competition display enhanced resistance to herbivory.

**Conclusions:**

This study provides a comprehensive list of genes whose expression is affected by intraspecific competition in *Arabidopsis*. The outcome is a unique response that involves genes related to light, nutrient deficiency, abiotic stress, and defence responses.

## Background

Due to their sessile nature, plants have constantly to adjust to their changing environment. Temperature fluctuations, variation in water content in soil, and pathogen attacks are some of the environmental factors with which plants have to cope. In particular, the presence of plant neighbours often reduces the availability of resources including light, water and nutrients. This struggle for common limited resources, which leads to decrease in growth, survival and fecundity, is defined as ‘competition’. Furthermore, since plants often have passive seed dispersal, competition can occur between plant neighbours from the same species (intraspecific competition) and, even more, from closely related individuals [1].

Competition does not only refer to the passive exploitation of limited resources by plants, but also to an active response to interferences caused by neighbours. It is now clearly established that plants are able to detect and interact with neighbours in different manners. Aboveground, the presence of neighbours can lead to a decrease in light intensity and quality available for the plant. Before light resource becomes limiting, plants exhibit morphological and growth responses in order to reach a more favourable light environment. The triggering signal of these responses is the detection by phytochrome photoreceptors of a low ratio of red to far-red radiation (R:FR), which is the consequence of selective absorbance of red light by neighbouring leaves [2]. The release of volatile organic compounds (VOCs) is also known to be an important parameter of plant-plant interactions [3]. For example, the phytohormone ethylene is a VOC overproduced by plants exposed to poor light quality and intensity. Accumulation of ethylene in the canopy was shown to participate in the shade avoidance response [4].

At the underground level, there is now consistent evidence that roots not only respond to nutrient availability but also to neighbouring roots. For example, *Pisum sativum*[5] can discriminate their own roots (self) from roots of a neighbouring plant (non-self). Several studies reported differential root growth when competitors were genetically related or non-related to the focal plant [6,7]. However, it is still debated if these responses are based on specific recognition processes or are the consequence of different competitive abilities [8,9].

How plants perceive conspecific neighbours and respond to competitive environment at the molecular level is still largely unknown. A first answer was provided by Schmidt and Baldwin [10] who studied transcriptional responses of *Solanum nigrum* to competition. Using a DNA microarray containing 568 genes, the authors showed that competition leads to important changes in gene expression, including genes mostly involved in stress-signalling and defence pathways. This work was the first to enlighten the molecular mechanisms underlying response of plants to competition. A recent study analyzed the genome-wide response to competition in *Arabidopsis* shoots and identified hundreds of genes that were up- and down-regulated, including genes involved in defense and photosynthesis [11]. However, both studies only investigated the transcriptional response to competition in leaves. To get more insight on the molecular mechanisms involved in competition, we conducted a complete transcriptome analysis of *Arabidopsis* plants subjected to intraspecific competition and analyzed gene expression changes in leaves and roots separately. Organ-specific transcriptome signatures were identified and revealed that competition activates genes related to nutrient starvation, biotic and abiotic stresses. In addition, we showed that larvae of the generalist herbivore *Spodoptera littoralis* performed better on plants subjected to competition than on single plants.

## Results

### Effect of intraspecific competition on performance of *Arabidopsis* plants

**Figure 1:**
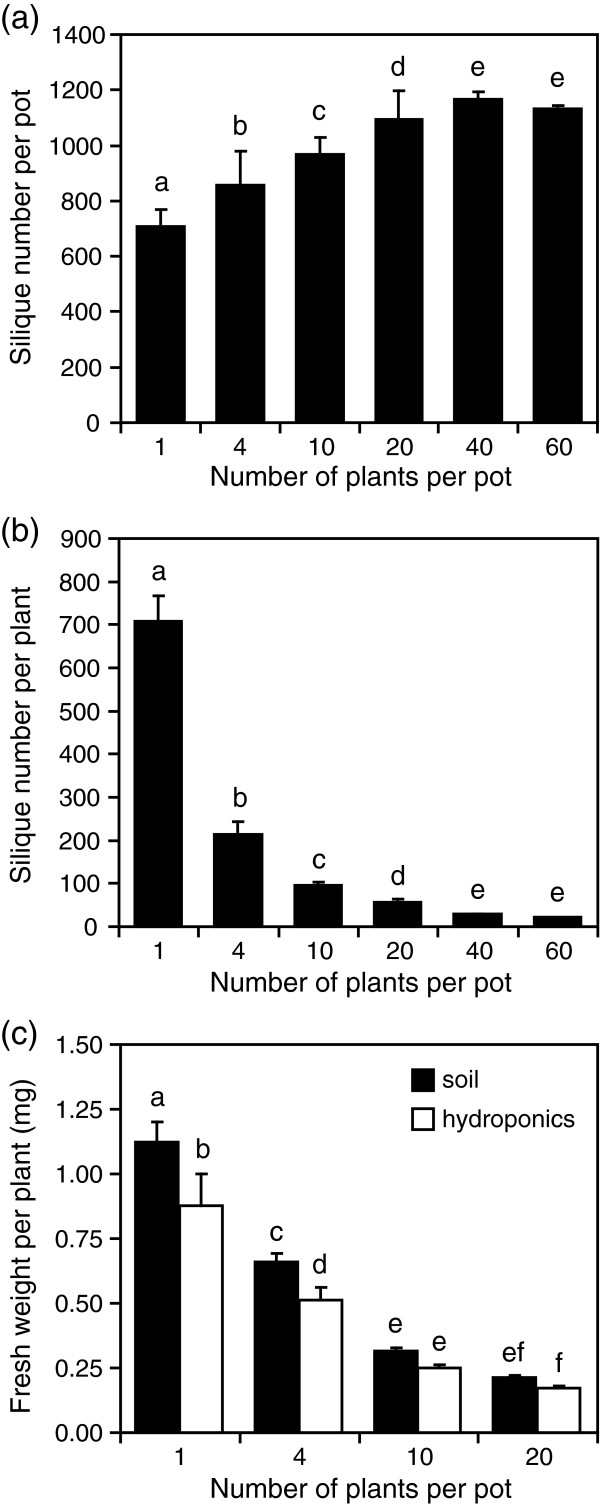
***Arabidopsis *performance under competition at different densities.** (**a**) Total number of siliques produced per pot. (**b**) Average number of siliques produced by a single plant. (**c**) Mean fresh weight per plant growing in soil or hydroponic culture (baked clay beads). Data are the mean (±SE) of three independent biological replicates. Different lowercase letters indicate significant differences (One-way ANOVA, Tukey HSD test, P < 0.05).

We investigated the consequence of intraspecific competition on fitness and survival of *Arabidopsis* plants growing at increasing densities of 1 to 60 plants per pot containing 380 cm^3^ of soil. This corresponded to a density of 156 to 9360 plants/m^2^, respectively. The total number of siliques produced in a pot increased significantly with increasing plant density, but reached a maximum at a density of 40 plants per pot (6420/m^2^) (Figure 1a). However, silique production per plant decreased significantly with increasing plant density (Figure 1b). Plant biomass was also affected by competition. The average fresh weight per plant also decreased significantly with increased density, with plants growing at 20 plants per pot (3120/m^2^) being 4-fold lighter than plants growing alone (Figure 1c).

We also monitored plant survival at the date of harvesting. A plant was considered a survivor if it was able to produce siliques. Survival was not significantly affected at densities below 10 plants per pot (1560/m^2^) and slightly affected at densities of 20 (survival 97%) and 40 plants (98%) per pot (data not shown). However, survival was reduced to 89% at a density of 60 plants per pot, showing that this density was too high to allow all plants to fully accomplish their cycle.

Since extracting RNA from roots grown in soil is often accompanied by a low recovery of intact root system and an inhibitory effect of soil particles on enzymatic reactions, we tested a hydroponic growth system as an alternative to soil. We selected baked clay beads, which constitute an inert material that keeps high moisture levels while being well aerated. We compared plant growth in soil and in baked clay beads at increasing densities. Overall, plants growing in hydroponic conditions were significantly lighter than plants growing in soil (Figure 1c). However, they were equally sensitive to competition and showed a significant reduction in biomass with increasing density. In addition, plant survival was similar in baked clay beads than in soil (not shown) and plants were able to accomplish their entire life cycle. These results demonstrate that this hydroponic system leads to similar competition responses than classical soil. We thus chose to perform a transcriptome analysis on plants grown in baked clay beads at a density of 20 plants per pot (3120/m^2^), which corresponded to a strong competition without death of individuals, and to compare it with plants growing without competition (1 plant per pot, 156/m^2^).

### Leaf and root transcriptome changes in response to competition

**Figure 2:**
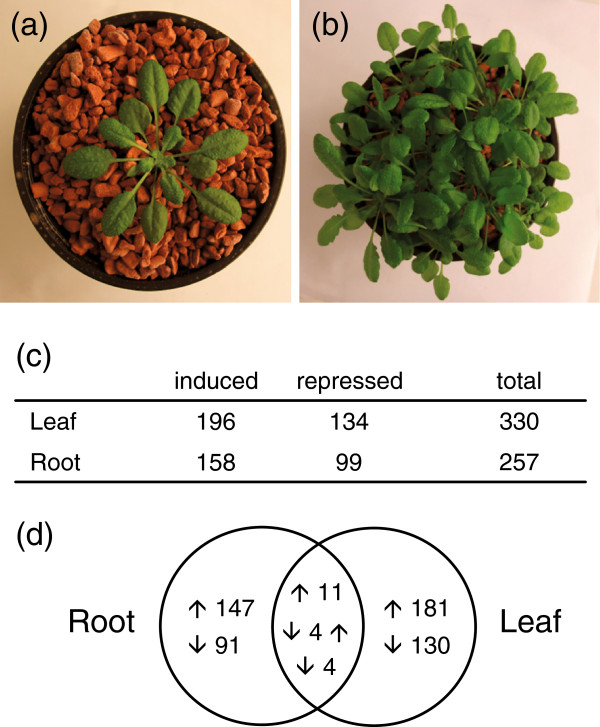
**Genes differentially expressed in response to competition. ***Arabidopsis* plants were either grown alone (**a**) or at a density of 20 plants (**b**) in hydroponic conditions in pots containing baked clay beads. Arabidopsis whole-genome microarrays were used to analyze the effect of competition on gene expression. Number of genes differentially regulated by competition in leaves and roots are presented (**c**). Venn diagram (**d**) gives the number of overlapping and non-overlapping differentially expressed genes. ↑, induced genes; ↓, repressed genes. All selected genes have an expression ratio ≥1.5 or ≤0.67 and a FDR <0.1.

To examine the effect of competition on gene expression, we extracted RNA from plants growing either alone or at a density of 20 plants per pot (Figure 2a,b). Leaf or root samples were hybridized to a microarray containing 22,473 gene-specific tags [12]. This experiment was repeated seven times independently to generate a robust list of differentially expressed genes and to be able to detect modest changes in gene expression with good probability. In leaves, 196 genes were induced and 134 genes were repressed, whereas in roots 158 genes were induced and 99 were repressed (Figure 2c). Interestingly, most differentially expressed genes were organ-specific. However, there were significantly more genes (19) than expected by chance that exhibited significant expression differences in both leaves and roots (Cumulative hypergeometric distribution, P = 1.6 × 10^-9^; Figure 2d; Additional file 1). This indicated that a small but significant part of the response to competition was similar between leaves and roots.

To validate the microarray data, we selected five genes differentially expressed in response to competition and performed a real-time quantitative PCR (qPCR) analysis on independent plant samples. Expression profiles were validated for all selected genes and for both organs, showing that qPCR results are in complete accordance with microarray results (see Additional file 2).

### Functional classification of competition-responsive genes

To identify key biological processes involved in response to competition, we performed a gene ontology (GO) enrichment analysis on differentially expressed genes. In leaves, the most significant functional groups included responses to abiotic stimulus, to chemical stimulus, to light stimulus (response to red or far red light, and response to light intensity) and to endogenous stimulus (see Additional file 3). Other enriched GO terms included responses to biotic stimulus and to other organisms. This analysis also revealed enrichment for biological processes related to nutrients and particularly to nitrogen metabolism.

In roots, the most significantly enriched GO term was “localisation”, which corresponds to any process in which a substance or a cellular entity is transported to and/or maintained in a specific location. Two enriched GO terms were related to nutrients: cation transport and sulphur compound metabolic process. Compared to leaves, “responses to stimulus” were categories that were also overrepresented in the list of differentially expressed genes in roots, although fewer categories were present. Furthermore, competition seemed to affect root secondary metabolism (see Additional file 3).

**Figure 3:**
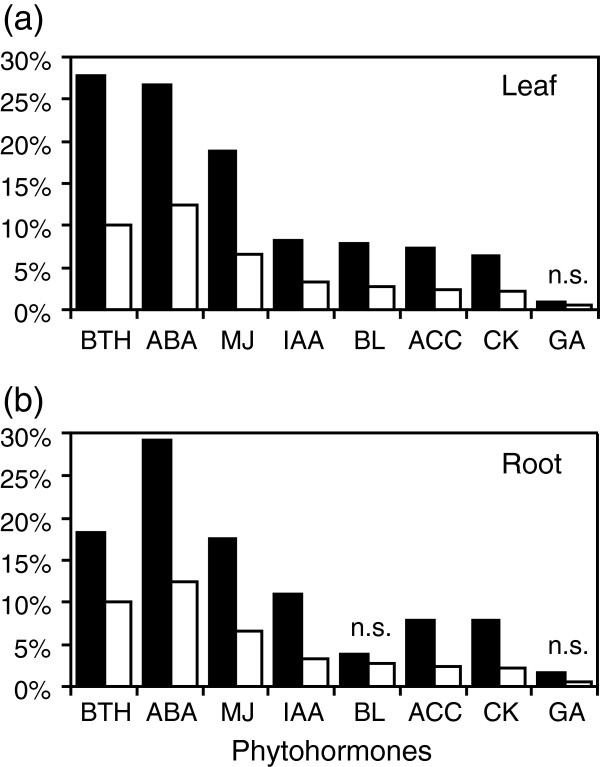
**Impact of competition on phytohormone-responsive genes.** The proportion of competition-responsive genes (black bars) in leaf (**a**) and root (**b**) that also respond to a given phytohormone treatment is shown. Values are compared with the proportion of phytohormone-responsive genes in the whole Arabidopsis genome (white bars). A Fisher’s exact test was applied to determine if each proportion is significantly different from its corresponding proportion in the whole genome (P < 0.001). Pairs that are not significantly different are labeled “n.s.” (not significant). Lists of hormone-responsive genes were obtained from Nemhauser et al. [13] and Wang et al. [14]. BTH, benzothiadiazole (salicylic acid analog); ABA, abscisic acid; MJ, methyl jasmonate; BL, brassinolide; IAA, indole-acetic acid; ACC, 1-aminocyclopropane-1-carboxylic-acid (ethylene precursor); CK, cytokinine; GA, gibberellic acid.

Phytohormones are key regulators of many biological processes, including growth and defence. To identify if phytohormones were involved in response to competition, we searched for significant enrichment of hormone-responsive genes obtained from the literature [13-15]. First, most phytohormone-responsive genes were over-represented in leaf and root expression data, showing that competition leads to an important biological reprogramming implying the action of plant hormones (Figure 3). The exception was gibberellin-responsive genes that were not significantly over-represented in competition-responsive genes, whereas brassinosteroid-responsive genes were only over-represented in the leaf dataset. Second, the three most enriched categories included genes that respond to the salicylic acid analogue benzothiadiazole (BTH), abscisic acid (ABA) and methyl jasmonate (MJ), which are hormones primarily involved in responses to biotic and abiotic stresses (Figure 3).

### Global analysis of the competition transcriptome by GSEA

Plants compete for many limiting resources at the same time (nutrients, water, space, light) and have to integrate several environmental cues. Consequently, the transcriptome response to competition might be difficult to interpret. To obtain a better insight in biological processes that underlie the response to competition, we performed a Gene Set Enrichment Analysis (GSEA) of the expression data. GSEA is a powerful analytical method that has the advantage of considering the whole transcriptome instead of a list of arbitrarily selected up- or down-regulated genes [16]. GSEA provides a normalized enrichment score (NES) that reflects whether members of a defined gene set occur toward the top (induced genes) or bottom (repressed genes) of a ranked list of genes from an experiment.

We selected conditions to which plants can be exposed in their environment, including nutrient starvation, abiotic stress, and biotic stress. Searching literature and microarary databases, we obtained transcriptome data from experiments were raw data were available and where the level of replication was sufficient to allow robust statistical analyses. For each of these datasets we established a list of up-regulated and down-regulated genes using similar selection criteria (log_2_ expression ratio ≥1 and ≤−1, FDR <0.05). Using these gene sets, we subjected leaf and root competition datasets to GSEA with the aim to find a correlation between competition and any of the selected conditions (see Methods).

**Figure 4:**
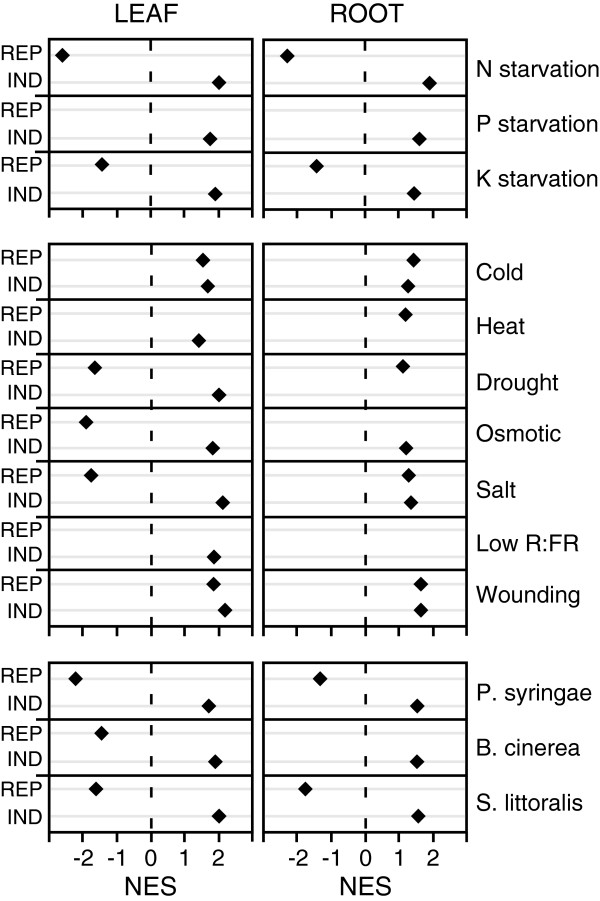
**Gene set enrichment analysis (GSEA) of competition microarray data.** List of genes that are highly induced (IND) or repressed (REP) in several biotic and abiotic conditions were obtained from published experiments or publicly available microarray data (see Methods). Overrepresentation of these gene sets in leaf and root data from the competition experiment was assessed by GSEA. Normalized enrichment scores (NES) for significantly enriched gene sets (p < 0.05, FDR <0.25) are shown (diamond). A positive NES score indicates that a gene set is enriched in the list of genes up-regulated by competition, whereas a negative NES score indicates that the gene set is enriched in the list of down-regulated genes.

Gene sets related to nutrient starvation were significantly enriched in leaf and root transcriptomes during competition. Genes influenced by N, P or K starvation were similarly influenced by competition (Figure 4). Only genes repressed by P starvation were not enriched in either leaf or root transcriptomes. These data suggest that competition triggers transciptome changes that are close, but not identical, to nutrient deficiency. In addition, no enrichment was detected for genes involved in iron and sulphur deficiency (not shown).

For conditions related to abiotic stresses, there was again a significant enrichment of gene sets in leaf and root transcriptomes (Figure 4). Strikingly, cold-, salt- and wound-responsive genes were all enriched in genes induced by competition in roots. In particular, genes normally repressed by these abiotic stresses were induced during competition. In leaves, genes induced or repressed by drought, osmotic stress, and salt stress, were enriched in genes induced or repressed by competition. Interestingly, cold- and wound-responding genes were enriched in genes induced by competition, whether they belonged to repressed or induced gene sets (Figure 4). Finally, there was a significant enrichment of genes induced by low R:FR ratio in genes induced by competition in leaves. Thus, plants subjected to competition showed similarities to plants exposed to several abiotic stresses at the transcriptome level but also displayed specific differences.

Finally, for gene sets related to biotic stresses, significant enrichments were found in root and leaf transcriptomes. Genes induced by *P*. *syringae*, *B*. *cinerea* and *S*. *littoralis* infestation were enriched in the lists of genes induced by competition, whereas genes repressed by these stresses were also repressed by competition, with the exception of B. cinerea set of repressed genes that was not enriched in genes that are downregulated by competition in roots (Figure 4). This shows that competition shares similarities with bacterial, fungal and herbivore attacks at the molecular level.

One limitation of this analysis was that many gene sets were obtained from experiments using very young plants (see Additional file 4). It is thus possible that we underestimated the number of gene sets enriched in the competition transcriptomes because of plant age.

### Detailed analysis of genes responding to competition

Competition altered the expression of several genes that play a role in mineral transport and metabolism. In leaves, several genes related to N assimilation were repressed (nitrate reductase (*NIA2*), nitrate reductase (*NIR1*), glutamate dehydrogenase (*GDH2*), glutamine synthase (*GS2*)), whereas *DUR3*, which encodes an urea/H+ symporter induced by nitrate starvation, was induced (Table 1). On the contrary, several ion transporters were induced in roots, including two nitrate transporters (*NRT2*.*2*, *NRT2*.*5*), *DUR3*, a P transporter (*PHTI*;*4*), and a potential K transporter (*CHX17*). A gene involved in allocation of K from root to shoot was repressed (*SKOR*). These results suggest that plants growing at high density react to competition for nutrients by up-regulating ion-transporters in the root and decreasing nutrient assimilation in the leaf.

**Table 1:** Selected genes regulated in response to competition

**Description**	**AGI code**	**Ratio (log**_**2**_**)**	**FDR**
**Leaf**			
*Mineral transport and metabolism*			
DUR3, urea transporter	At5g45380	0.79	0.034
GS2, glutamine synthetase	At5g35630	−0.72	0.089
GDH2, glutamate dehydrogenase	At5g07440	−0.74	0.043
NIR1, ferredoxin-nitrate reductase	At2g15620	−1.07	0.021
NIA2, nitrate reductase	At1g37130	−1.35	0.021
*Biotic stress response*			
HPL1, hydroperoxide lyase	At4g15440	1.60	0.079
JR1, jacalin lectin	At3g16470	1.43	0.018
PDF1.1, defensin	At1g75830	1.00	0.093
PR-4, hevein-like	At3g04720	0.87	0.011
disease resistance protein (TIR-NBS)	At1g72940	0.86	0.080
THI2.2, thionin	At5g36910	−0.68	0.014
pathogenesis-related thaumatin	At1g20030	−1.24	0.016
*Abiotic stress response*			
ERD1, early response to dehydration	At5g51070	0.80	0.071
HSP70, heat shock protein	At3g12580	0.74	0.020
DI19, drought-induced protein	At1g56280	0.67	0.063
*Shade avoidance response*			
XTR7, xyloglucan endotransglycosylase	At4g14130	2.03	0.021
HFR1, transcription factor	At1g02340	1.57	0.031
HAT2, transcription factor	At5g47370	1.32	0.012
PHYA, phytochrome A	At1g09570	0.84	0.021
FHL, phyA nuclear import	At5g02200	0.79	0.095
ASA1, ubiquitin-protein ligase (auxin transport)	At3g02260	0.59	0.071
*Hormone metabolism and signalling*			
IAA29, transcription factor	At4g32280	1.79	0.022
SAUR-like auxin-responsive protein	At1g56150	1.15	0.052
BGL1, ABA-glucoside hydrolase	At1g52400	0.84	0.070
BR6OX2, brassinosteroid oxidase	At3g30180	0.79	0.048
ARF2, transcription factor	At5g62000	0.76	0.093
EIN2, ethylene signal transduction	At5g03280	0.67	0.030
AUX1, auxin influx transporter	At2g38120	0.62	0.012
ABA1, zeaxanthin epoxidase	At5g67030	−0.59	0.043
CKX4, cytokinin dehydrogenase	At4g29740	−0.79	0.041
GA4, gibberellin 3-beta-dioxygenase	At1g15550	−0.86	0.034
*Secondary metabolism*			
F3H, naringenin 3-dioxygenase (flavonoid)	At3g51240	−0.61	0.081
CER1, aldehyde decarbonylase (wax)	At1g02205	−0.61	0.048
farnesyltransferase (terpene)	At3g11950	−0.62	0.081
SMT3, sterol methyltransferase (sterol)	At1g76090	−0.70	0.026
fatty acid condensing enzyme (wax)	At2g16280	−0.75	0.082
CHS, chalcone synthase (flavonoid)	At5g13930	−1.42	0.016
*Photosynthetic activity*			
fructose-bisphosphate aldolase	At2g21330	−0.59	0.014
GAPB, glyceraldehyde-3P dehydrogenase	At1g42970	−0.61	0.065
PSB28, (photosystem II reaction center)	At4g28660	−0.68	0.070
**Root**			
*Mineral transport and metabolism*			
NRT2.5, nitrate transporter	At1g12940	4.55	0.002
DUR3, urea transporter	At5g45380	2.50	0.010
NRT2.2, nitrate transporter	At1g08100	2.02	0.062
PHT1;4, phosphate transporter	At2g38940	0.76	0.063
CHX17, cation:proton antiporter	At4g23700	0.93	0.014
AMT1;3, ammonium transporter	At3g24300	0.63	0.034
SKOR, outward rectifier potassium channel	At3g02850	−1.22	0.043
NIR1, ferredoxin-nitrate reductase	At2g15620	−1.26	0.014
NRT2.6, nitrate transporter	At3g45060	−3.00	0.002
*Biotic stress response*			
ERF/AP2 transcription factor	At5g51190	2.38	0.043
CYP81F2 (glucosinolates)	At5g57220	1.55	0.070
defensin-related	At3g63360	1.39	0.062
protease inhibitor	At3g22600	1.21	0.042
JAZ4, jasmonate repressor	At1g48500	1.01	0.074
chitinase	At2g43590	0.85	0.095
BAT5, bile:acid sodium symporter (glucosinolates)	At4g12030	0.72	0.039
disease resistance protein (TIR class)	At4g19925	0.66	0.063
BCAT4, branched-chain aminotransferase (glucosinolates)	At3g19710	0.59	0.077
chitinase	At4g19750	−1.57	0.037
*Abiotic stress response*			
HSF4, heat-shock transcription factor	At4g36990	0.91	0.043
RbohE, NADPH oxidase	At1g19230	0.74	0.052
dehydrin	At4g38410	−0.59	0.090
COR78, cold-regulated protein	At5g52310	−0.69	0.063
osmotin-like protein	At2g28790	−0.90	0.030
*Hormone metabolism and signalling*			
auxin efflux carrier	At2g17500	1.33	0.024
ABF3, ABA-responsive transcription factor	At4g34000	0.77	0.038
EFE, ethylene forming enzyme	At1g05010	0.66	0.090
ACC oxidase (ethylene biosynthesis)	At3g47190	0.60	0.094
ERS2, ethylene receptor	At1g04310	−0.67	0.070
*Secondary metabolism*			
UGT72E1, coniferyl-alcohol glucosyltransferase (lignin)	At3g50740	0.97	0.064
HSD4, hydroxysteroid dehydrogenase	At5g50590	0.95	0.010
HCT, hydroxycinnamoyl transferase (lignin)	At5g48930	0.71	0.025
CAD4, cinnamyl-alcohol dehydrogenase (lignin)	At3g19450	0.69	0.065
FAH1, ferulate 5-hydroxylase (lignin)	At4g36220	0.67	0.070
4CL2, 4-coumarate-CoA ligase (lignin)	At3g21240	0.59	0.053

Genes differentially expressed by competition were selected from the microarray data. Ratios (log_2_) were calculated by comparing gene expression in plants growing at a density of 20 plants/pot with gene expression in plants growing at a density of one plant/pot. Values are the average of seven independent experiments.

Genes typically involved in different defence pathways were found in the list of up-regulated genes in leaves and roots (Table 1). This included *JR1*, *HPL*, and glucosinolate biosynthesis genes (*CYP81F2*, *BAT5*, *BCAT4*), which have been shown to be regulated by the jasmonate pathway during attacks by necrotroph fungi, wounding or herbivory [17,18]. This list also contained PR-4 and disease resistance proteins (At1g72940, At2g14080, At4g19925), which participate in the response to biotroph bacterial pathogens that is controlled by the salicylic acid pathway ( http://www.genevestigator.com, [15]). These findings indicate that plants undergoing competition might anticipate potential enemies by activating defence mechanisms. In addition, competition triggered the expression of abiotic stress-related genes that are often associated with biotic stresses. These included genes involved in drought stress (*ERD1*, *DI19*), heat stress (*HSP70*, *HSF4*), and oxidative stress (*RbohE*) (Table 1).

Plant hormones regulate numerous growth and development processes, as well as responses to stresses. We showed that plants growing at a density of 20 plants per pot had a four-fold smaller biomass than plants growing alone (Figure 1). This was correlated with a differential expression of many genes involved in hormone biosynthesis or signalling (Table 1). For instance, several genes modulating auxin transport and signalling (*IAA29*, *SAUR*-*like*, *IAA2*, *ARF2*, *AUX1*), and brassinosteroid biosynthesis (*BR6OX2*) were up-regulated by competition in leaves, whereas genes involved in ABA (*ABA1*), cytokinin (*CKX4*), and gibberellin (*GA4*) biosynthesis were repressed. In roots, ethylene biosynthesis genes were up-regulated (*EFE*, *ACC oxidase*). In addition, we identified several genes known to respond to low R:FR ratio (Table 1). These genes are either involved in light signalling (*HFR1*, *PHYA*, *FHL*), in auxin-regulated elongation processes (*HAT2*, *XTR7*), or in auxin transport (*ASA1*), suggesting that plants growing at high density detected a change in light quality and triggered a growth response modulated by auxin.

Plants responded to competition by down-regulating secondary metabolism in leaves. Genes controlling flavonoid (*CHS*, *F3H*, isoflavone reductase), sterol (*SMT3*), terpene (farnesyltransferase), or wax (*CER1*) biosynthesis were repressed (Table 1). Interestingly, several genes encoding enzymes of lignin biosynthesis (*HCT*, *CAD4*, *FAH1*, *4CL2*) and a R2R3-MYB transcription factor (*MYB48*) known to be involved in lignin biosynthesis [19] were up-regulated in roots, suggesting a stimulation of secondary growth during competition (Table 1 and Additional file 1).

Many species have been shown to change behaviour and pattern of growth in response to changes in local density. For example, bacteria produce several types of signalling molecules allowing them to coordinate gene expression according to the density of their local population. We searched for competition-specific genes in our microarray data. The rationale was to identify genes that are specifically associated with plant-plant interactions and that do not respond to indirect effects caused by competition. For this purpose, we searched for genes that responded to competition in our experiments but did not exhibit changes in expression in the multiple treatments or conditions that are stored in Genevestigator microarray database ( http://www.genevestigator.com; [15]). Results of this analysis revealed that nearly all our competition-responsive genes were also affected by at least another condition. Interestingly, one gene encoding a putative hydroxysteroid dehydrogenase (*HSD4*, At5g50590) was not differentially expressed in the many conditions previously tested, and thus apparently responded only to competition. HSD4 was induced by competition in roots but not in leaves (Table 1 and Additional file 1) and this differential expression was confirmed by qPCR on independent RNA samples (see Additional file 2). HSD1, a close homologue of HSD4, was shown to play a role in growth and development [20]. Therefore, HSD4 provides an interesting candidate gene to further study processes directly linked to competition.

The list of 19 genes differentially regulated in both leaves and roots did not reveal any conserved physiological process but, interestingly, there were five transcription factors (HAP2B, BEL1, MYB111, NAP and a C3HC4 zinc finger protein) among these genes (Additional file 1).

### *Spodoptera littoralis* show lower performance on plants subjected to competition

**Figure 5:**
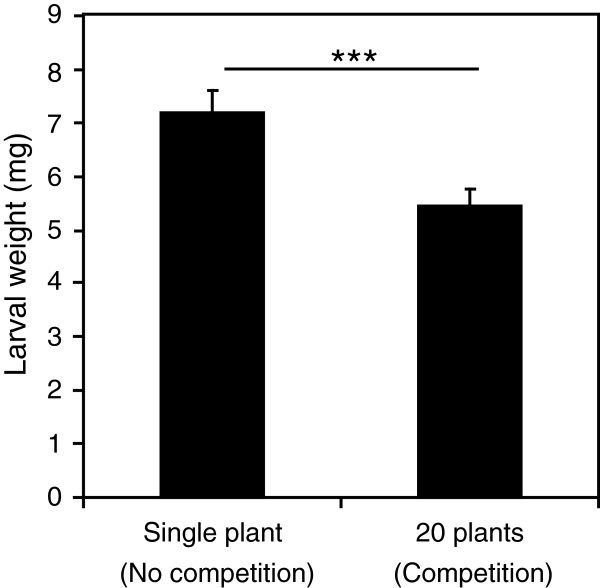
**Effect of *Arabidopsis *intraspecific competition on insect larval performance.** Freshly hatched larvae of the generalist *Spodoptera littoralis* were placed on plants growing alone or at a density of 20 plants per pot. Larval weight was measured after eight days of feeding. Values are the mean (±SE) of three independent biological replicates.

Competition induced many genes related to defence and our GSEA analysis showed a positive enrichment of genes induced by biotic stresses, including herbivory by *S*. *littoralis*. We thus postulated that plants growing in a competitive environment might be prepared to insect attack. To test this hypothesis, we challenged *Arabidopsis* plants growing alone in a pot or at a density of 20 plants per pot with neonate *S*. *littoralis* larvae. Strikingly, we found that larvae feeding for 7 days on plants undergoing competition were significantly smaller that those feeding on single plants (Figure 5). These results show that plants growing in competition lead to lower insect performance.

## Discussion

Despite the large number of studies about plant competition, our understanding of the genetic and molecular basis for competition is still meager. In this study, we showed that intraspecific competition in *Arabidopsis* alters the expression of 330 genes in leaves and 257 genes in roots. Since we collected all plants in a pot these numbers might be underestimated due to the fact that edge plants might not experience the same conditions or signals than center plants, hence diluting expression ratios. Removing edge plants might reveal additional genes that strictly respond to plant density and competition. This would easily be done with aboveground parts but would be more difficult with roots that are intricately interlaced between center and edge plants.

Genes regulated by competition appeared to belong to very different pathways or categories. This diversity clearly reflects the nature of competition, which is a combination of several individual stresses and conditions. Using GSEA, we observed a typical response to N, P and K deficiency in plants growing at high density and several N, P and K transporter genes were induced by competition. This could be interpreted as a response to a general nutrient starvation, as these transporter genes are known to be induced by deficiency in the nutrient that they transport, and could logically explain part of the decrease of fresh weight observed for plants growing at high density.

Aboveground, competition for light in dense canopies leads to a decrease in light intensity but also in quality that is reaching leaves. Plant phytochrome photoreceptors react to variations in R:FR ratio by maximizing growth and orientation, a phenomenon called shade avoidance syndrome (SAS). Numerous studies on the mechanisms and transcriptional responses to SAS revealed that growth changes associated with SAS require auxin [2,21,22]. Recently, it was shown that low R:FR ratio changed the cellular location of the modulator of auxin efflux PIN-FORMED 3 (PIN3), leading to increased hypocotyl growth. Interestingly, *Arabidopsis pin3* mutants were outcompeted by wild-type plants in mixed culture, demonstrating the importance of SAS and auxin during competition [23]. Accordingly, we observed a positive enrichment of the gene set induced by shade avoidance response in leaves, including genes responding to auxin, indicating that competition was triggering SAS. Although our experiments were carried-out in a growth chamber with fluorescent lights that provide a higher R:FR ratio than in natural conditions, the observation that SAS genes were induced in plants growing at high density suggests that changes in light quality were anyhow detected by these plants. This might indicate that the variation in R:FR ratio at the canopy level in densely grown plants was sufficient to change phytochrome photoequilibrium and to trigger downstream expression changes. These changes might be amplified in a natural light environment where variations in R:FR ratios are more drastic. Further experiments to test the effect of competition on SAS gene expression in nature will be interesting. In addition, genes reported to be repressed during SAS were not significantly altered in our experiments. This partial shade avoidance response could be due to a modulation of the classical shade avoidance response by other factors. In leaves, other components like VOCs, including ethylene, could modulate SAS [3,4]. Alternatively, SAS occurring in leaves could be adjusted by a root-to-shoot signalling in response to underground competition.

Competition modified the expression of many genes involved in abiotic and biotic stresses, both in roots and leaves. It was shown previously that competition alters the expression of many stress-responding genes in *S*. *nigrum*[10]. A study on *Centaurea maculosa* plants demonstrated that defence-related secondary metabolites accumulate in plants under intraspecific competition [24]. However, we found that the overall profile of gene expression in response to competition did not correspond to a typical profile that can be attributed specifically to a single stress. Instead, we observed a signature that resulted from several inputs. It is known that deficiency in certain nutrients can induce responses related to abiotic stress or defence. For example, K and N starvation lead to the activation of some defence genes dependant on jasmonate [25,26], whereas iron starvation triggers the expression of the salicylic acid-dependent genes [27]. The transcriptional response to a combination of drought and heat consisted of a unique signature that included only some genes of the two individual stresses [28]. Similarly, the transcriptional response to competition is an original response corresponding to the integration of different environmental cues.

A striking feature of the effect of competition was the enrichment of genes that respond to herbivory and to bacterial and fungal infections. This raises the question whether this enrichment underlines a biotic response due to plant-plant interactions or whether it is an indirect consequence of nutrient deficiency, reduced growth or changes in light quality. Reports of root discrimination [5] and kin recognition [6,7] tend to support the hypothesis that there is a detection process among competing plants. However, we were unable to provide any evidence for a specific recognition between kin and non-kin plants in *Arabidopsis*[9]. In addition, no root discrimination phenomenon has yet been reported in *Arabidopsis*. More studies will be necessary to better understand the regulation of defence gene expression in the context of competition.

Our results showed a decreased performance of caterpillars feeding on *Arabidopsis* plants subjected to competition. Although we cannot rule out that this effect is due to a lower nutritional value of plants growing at high density, a lack of induction of primary metabolism genes and, on the contrary, the upregulation of defence genes may be interpreted as an enhanced resistance of these plants. Such a positive effect of competition on defence, described as the “Defence Stress Benefit Hypothesis”, was reported in some studies [29,30]. However, another report showed that aphids and leaf miners were more abundant on plants subjected to competition [31], whereas Agrawal [32] found no effect of competition on defence metabolite accumulation or herbivory. In *Arabidopsis*, *S*. *frugiperda* larvae were bigger on plants growing in high density than on plants growing in low density [33]. However, in the last example, plants were grown in individual pots and no competition took place in the rhizosphere. On the contrary, ecological studies showed that plants in low nutrient environments grow slowly, invest in constitutive defences and are less affected by herbivory than plants growing in rich nutrient environments [34]. Similarly, it was recently shown that *Arabidopsis* plants responded to K-deficiency by upregulating jasmonate-dependent genes and that they were more resistant to thrips attack [35]. Thus, the outcome of competition on defence seems to depend on the species, the neighbours’ identity, the environmental conditions or the nature of the attacker. Altogether, these different results argue for a complex and coordinated response to competition, which might be in some cases beneficial for defence against pathogens. It would be interesting to test whether the induction of defence genes in response to competition and the reduced larval performance is only occurring when *Arabidopsis* plants compete with their kin or whether it also occurs under interspecific competition. In addition, densely grown plants might create a humid micro-environment that would favor infection by fungal or microbial pathogens. It would thus be interesting to know if induction of defence genes by competition plays a role in other biotic stresses, including fungal or bacterial infections.

## Conclusions

In conclusion, this study reveals that response to competition at the molecular level is the result of the integration of different signals originating from aboveground and belowground levels. The outcome is a unique response that involves genes related to light, nutrient deficiency, abiotic stress, and defence responses. Results from this work provide a comprehensive map of genes whose expression is affected by intraspecific competition in *Arabidopsis*. Future studies should focus on the precise roles of competition-responsive genes in plant-plant interactions and on the effect of competition on resistance to subsequent stresses and pathogen attacks.

## Methods

### Plant material and growth conditions

*Arabidopsis thaliana* ecotype Columbia (Col-0) was used in all experiments. Seeds were surface-sterilized by immersion in 80% ethanol and 0.4% sodium hypochlorite solution for 10 minutes, followed by 4 rinses with 95% ethanol and allowed to dry on a sterile filter. Dried seeds were sown on plates containing Â½ Murashige and Skoog medium (MS; Sigma) with 0.25% sucrose and 0.8% phytoagar (Sigma). Seeds were exposed to a 3-day stratification treatment in a cold dark room at 4°C before transfer to a climate chambers at 22°C, with a 16 h/8 h photoperiod, 120 μmol photons m^−2^ · s^−1^ provided by Osram Lumilux L58W/830 Warm White lamps (for light specifications, see Additional file 5), and 50% relative humidity. One-week old seedlings were transplanted in 9-cm diameter pots (volume of 380 cm^3^) containing either soil for standard culture or baked clay beads for hydroponic culture. Plants were grown in growth chambers at 22°C, with a 16 h/8 h photoperiod, 120 μmol photons m^−2^ · s^−1^, and 50% relative humidity.

For hydroponic culture, pots were filled with baked clay beads (Ton-granulat, Seramis, http://www.seramis.de). Freshly transplanted seedlings were moistened every day with a water spray and kept under a transparent cover for 5 days. Every 4 days, pots were drenched alternately with water or with a fertilizer solution for hydroponics (0.57 mM NH_4_NO_3_, 0.182 mM P_2_O_5_, 0.212 mM K_2_O, and traces (<20 μM) of MgO, S, Fe, Mn, B, Cu, Na, Cl, Mo and Zn; Wuxal Hydro, Maag/Syngenta, http://www.syngenta-agro.ch). The excess solution was removed. After 2 weeks of culture, the watering solution was supplemented once with Fe-Sequestrene (0.13 mM Fe-ethylenediamine-N,N'-bis(2-hydroxyphenylacetic acid), 1.6 mM K2O, 0.4 mM NH_4_NO_3_; Sequestrene Rapid, Maag, http://www.maag-profi.ch).

### Competition experiments

One-week old seedlings were transplanted in soil or baked clay beads at different densities, ranging from 1 plant per pot (156 plants/m^2^) to 60 plants per pot (9360 plants/m^2^). Seedlings were placed evenly at the surface to ensure similar competition between plants. Pots were arranged randomly in trays to minimize position effects. For measurement of silique number, plants were grown until completion of their life cycle (ca. 2 months). For fresh weight measurement, plants were grown for 30 days.

### Microarray hybridization and data analysis

Plants were grown hydroponically for 40 days, at a density of one or 20 plants per pot. Plants were removed from the pots and quickly washed with water to remove excess of beads attached to the roots. Leaves and roots from four pots (density 1) and one pot (density 20) were then collected separately and immediately stored in liquid nitrogen. RNA was extracted, reverse-transcribed, and labelled with Cy3- or Cy5-dCTP as previously described [18]. Labelled cDNA was hybridized to Complete Arabidopsis Transcriptome MicroArray (CATMA) microarrays containing 22'473 gene-specific tags [12]. Microarray hybridizations were performed with a dye-swap design. Scanning, normalization, and data analyses have been described previously [18]. To address the issue of multiple comparisons we calculated an FDR using a method developed for genome-wide studies [36]. Genes differentially expressed during competition were selected based on a threshold of 1.5-fold change and a FDR <0.1. Data are from seven independent biological replicates. Microarray data have been deposited in ArrayExpress ( http://www.ebi.ac.uk/arrayexpress/) under accession number E-MEXP-3735.

### Quantitative PCR analysis

Independent plant samples were prepared from plants growing hydroponically at a density of one or 20 plants per pot. Total RNA was prepared from leaves and roots from four pots (density 1) and one pot (density 20) using RNeasy® Plant Mini kit including DNase I on-column digestion according to the manufacturer’s instructions (Qiagen, http://www.qiagen.com). For cDNA synthesis, 1 μg of total RNA was reverse-transcribed using SuperScript® VILO™ cDNA Synthesis Kit (Invitrogen, http://www.invitrogen.com) in a final volume of 20 μl and cDNA was diluted 5 times in nuclease-free water. Quantitative real-time PCR analysis was performed in a final volume of 20 μl containing 1 μl of diluted cDNA, 0.2 μM of each primer, and 1X qPCR mastermix plus for SYBR® Green I (Eurogentec, http://www.eurogentec.com). Reactions were performed with the following thermal cycling program: 2 min at 50°C, 10 min at 95°C, 40 times cycling for 15 s at 95°C, 20 s at 55°C and 1 min at 72°C. Primer efficiencies were established by serial dilutions of an amplicon for each gene. Relative mRNA abundance was normalized to the geometric mean of the relative abundance of two references genes (SAND family protein, At2g28390; GAPDH, At1g13340). For primers used for this study, see Additional file 6. The experiment was done three times independently.

### Data processing and analysis

GO enrichment analysis was performed with AgriGO singular enrichment analysis using hypergeometric test [37]. All other parameters were set to default and the *Arabidopsis* genome was used as background.

For Gene Set Enrichment Analysis (GSEA), expression data related to nutrient starvation, abiotic and biotic stresses were collected from literature and publicly available microarray data. For each dataset, analysis was carried-out using an interface developed at the University of Lausanne (Gene Expression Data Analysis Interface (GEDAI)) [38]. Differentially expressed genes were identified by fitting a linear model for each gene and evaluating the fold change and moderated t-statistics P-values. P-values were corrected for multiple testing using the FDR method of Storey and Tibshirani [36]. Lists of significantly regulated genes (log2 ratio ≥1 and ≤−1, FDR <0.05) were ordered according to their mean log-fold change, allowing to define a top-ranked gene subset of induced genes (maximum 100 genes) and a top-ranked gene subset of repressed genes (maximum 100 genes). To establish gene sets representative of each condition, ranked gene lists were combined into a core list of significantly induced genes and a core list of significantly repressed genes (for detailed gene lists, see Additional file 4). Then, genes from the root and leaf transcriptome data were ranked according to their mean log-fold change. Root or leaf microarray datasets were subjected to GSEA to test whether gene sets from the different conditions were significantly enriched in the data. GSEA was performed with the GSEA-P desktop application using the “GseaPreranked” tool [39]. Enrichment scores were calculated using weighted enrichment statistic, and significance levels calculated by applying 2500 permutations. Normalized enrichments score (NES) considered to be significant had a nominal p-value <0.05 and a FDR q-value <0.25.

### Insect bioassay

*Spodoptera littoralis* (Egyptian cotton worm) eggs were obtained from Syngenta (Stein, Switzerland) and were stored at 10°C until further use. Eggs were incubated in a beaker covered with plastic film in a growth chamber (22°C, 65% relative humidity, 100 μmol m^-2^ sec^-1^, 10 h light/14 h dark photoperiod) to allow hatching. Plants were grown at a density of one or 20 plants per pot during 40 days. Eight pots (density 1) or two pots (density 20) were placed in transparent plastic boxes and 25 freshly hatched *S*. *littoralis* larvae were dispersed on plant leaves in each box. Larvae were able to move freely between pots and leaf material was in excess throughout the experiment. After 7 days, larval weight was measured with a precision balance. This experiment was repeated three times.

## Competing interests

The authors declare that they have no competing interests.

## Authors' contributions

FGM and PR conceived the study, designed the research and coordinated the project. FGM carried out the competition experiments, the molecular genetic studies and performed the statistical analyses. FB, FS and CG-D participated in growing plants, in carrying out microarray experiments and in performing bioassays. FGM, LK and PR were responsible for drafting and revising the manuscript with contributions from the co-authors. All authors read and approved the final manuscript.

## Supplementary Material

Additional file 1 **List of genes induced or repressed by competition.** This Excel table contains a list of genes significantly regulated by competition in *Arabidopsis* leaves. Genes have an expression ratio (log_2_) ≥0.59 or ≤−0.59 and a FDR <0.1. Click here for file

Additional file 2 **Validation of microarray data by qPCR.** Validation of microarray data by qPCR. Expression of selected genes differentially expressed in leaf and root tissues was analyzed on independent samples by qPCR. Expression ratios are calculated from experiments comparing plants growing at a density of 20 plants per pot and plants growing alone. Values (±SE) are normalized to the reference genes and are the mean of three biological replicates. Microarray data are shown for comparison. Click here for file

Additional file 3 **Gene ontology (GO) analysis of differentially expressed genes.** Enrichment of differentially expressed genes in different gene ontology (GO) categories. Genes induced or repressed by competition in both leaf or root samples were classifed in different GO categories with AgriGO singular enrichment analysis. Click here for file

Additional file 4 **List and origin of gene sets used for GSEA analysis.** This Excel table includes a list of genes used for GSEA analysis. Genes significantly induced (IND) or repressed (REP) in response to different treatments or conditions were extracted from the literature or from microarray databases. Source and description of published data sets are given as a separate Excel sheet. Click here for file

Additional file 5 **Lamp specifications.** Spectral composition of the Osram Lumilux L58W/830 Warm White lamps. Click here for file

Additional file 6 List of primers used for qPCR analysis. Click here for file

## References

[B1] Novoplansky A Picking battles wisely: plant behaviour under competition Plant Cell Environ 2009 32 726 741 10.1111/j.1365-3040.2009.01979.x 19389051

[B2] Franklin KA Shade avoidance New Phytol 2008 179 930 944 10.1111/j.1469-8137.2008.02507.x 18537892

[B3] Kegge W Pierik R Biogenic volatile organic compounds and plant competition Trends Plant Sci 2010 15 126 132 10.1016/j.tplants.2009.11.007 20036599

[B4] Pierik R Whitelam GC Voesenek LACJ de Kroon H Visser EJW Canopy studies on ethylene-insensitive tobacco identify ethylene as a novel element in blue light and plant-plant signalling Plant J 2004 38 310 319 10.1111/j.1365-313X.2004.02044.x 15078333

[B5] Falik O Reides P Gersani M Novoplansky A Self/non-self discrimination in roots J Ecol 2003 91 525 531 10.1046/j.1365-2745.2003.00795.x

[B6] Dudley SA File AL Kin recognition in an annual plant Biol Lett 2007 3 435 438 10.1098/rsbl.2007.0232 17567552 PMC2104794

[B7] Milla R Forero DM Escudero A Iriondo JM Growing with siblings: a common ground for cooperation or for fiercer competition among plants? Proc Royal Soc B: Biol Sci 2009 276 2531 2540 10.1098/rspb.2009.0369 PMC2686667 19403541

[B8] Biedrzycki ML Bais HP Kin recognition in plants: a mysterious behavior unsolved J Exp Bot 2011 61 4123 4128 20696656 10.1093/jxb/erq250

[B9] Masclaux F Hammond RL Meunier J Gouhier-Darimont C Keller L Reymond P Competitive ability not kinship affects growth of Arabidopsis thaliana accessions New Phytol 2010 185 322 331 10.1111/j.1469-8137.2009.03057.x 19886895

[B10] Schmidt DD Baldwin IT Transcriptional responses of Solanum nigrum to methyl jasmonate and competition: a glasshouse and field study Funct Ecol 2006 20 500 508 10.1111/j.1365-2435.2006.01122.x

[B11] Geisler M Gibson DJ Lindsey KJ Millar K Wood AJ Upregulation of photosynthesis genes, and down- regulation of stress defense genes, is the response of Arabidopsis thaliana shoots to intraspecific competition Bot Stud 2012 53 85 96

[B12] Allemeersch J Durinck S Vanderhaeghen R Alard P Maes R Seeuws K Bogaert T Coddens K Deschouwer K Van Hummelen P Vuylsteke M Moreau Y Kwekkeboom J Wijfjes AH May S Beynon J Hilson P Kuiper MT Benchmarking the CATMA microarray. A novel tool for Arabidopsis transcriptome analysis Plant Physiol 2005 137 588 601 10.1104/pp.104.051300 15710687 PMC1065359

[B13] Nemhauser JL Hong F Chory J Different plant hormones regulate similar processes through largely nonoverlapping transcriptional responses Cell 2006 126 467 475 10.1016/j.cell.2006.05.050 16901781

[B14] Wang D Amornsiripanitch N Dong X A genomic approach to identify regulatory nodes in the transcriptional network of systemic acquired resistance in plants PLoS Pathog 2006 2 e123 10.1371/journal.ppat.0020123 17096590 PMC1635530

[B15] Zimmermann P Hirsch-Hoffmann M Hennig L Gruissem W GENEVESTIGATOR. Arabidopsis microarray database and analysis toolbox Plant Physiol 2004 136 2621 2632 10.1104/pp.104.046367 15375207 PMC523327

[B16] Subramanian A Tamayo P Mootha VK Mukherjee S Ebert BL Gillette MA Paulovich A Pomeroy SL Golub TR Lander ES Mesirov JP Gene set enrichment analysis: a knowledge-based approach for interpreting genome-wide expression profiles Proc Nat Acad Sci USA 2005 102 15545 15550 10.1073/pnas.0506580102 16199517 PMC1239896

[B17] de Vos M Van Oosten VR Van Poecke RMP Van Pelt JA Pozo MJ Mueller MJ Buchala AJ Métraux J-P Van Loon LC Dicke M Pieterse CMJ Signal signature and transcriptome changes of Arabidopsis during pathogen and insect attack Mol Plant Microbe Interact 2005 18 923 937 10.1094/MPMI-18-0923 16167763

[B18] Reymond P Bodenhausen N Van Poecke RM Krishnamurthy V Dicke M Farmer EE A conserved transcript pattern in response to a specialist and a generalist herbivore Plant Cell 2004 16 3132 3147 10.1105/tpc.104.026120 15494554 PMC527203

[B19] Oh S Park S Han K-H Transcriptional regulation of secondary growth in Arabidopsis thaliana J Exp Bot 2003 54 2709 2722 10.1093/jxb/erg304 14585825

[B20] Li F Asami T Wu X Tsang EWT Cutler AJ A putative hydroxysteroid dehydrogenase involved in regulating plant growth and development Plant Physiol 2007 145 87 97 10.1104/pp.107.100560 17616511 PMC1976581

[B21] Carabelli M Possenti M Sessa G Ciolfi A Sassi M Morelli G Ruberti I Canopy shade causes a rapid and transient arrest in leaf development through auxin-induced cytokinin oxidase activity Genes Dev 2007 21 1863 1868 10.1101/gad.432607 17671088 PMC1935025

[B22] Tao Y Ferrer J-L Ljung K Pojer F Hong F Long JA Li L Moreno JE Bowman ME Ivans LJ Cheng Y Lim J Zhao Y Ballaré CL Sandberg G Noel JP Chory J Rapid synthesis of auxin via a new tryptophan-dependent pathway is required for shade avoidance in plants Cell 2008 133 164 176 10.1016/j.cell.2008.01.049 18394996 PMC2442466

[B23] Keuskamp DH Pollmann S Voesenek LACJ Peeters AJM Pierik R Auxin transport through PIN-FORMED 3 (PIN3) controls shade avoidance and fitness during competition Proc Nat Acad Sci USA 2010 107 22740 22744 10.1073/pnas.1013457108 21149713 PMC3012496

[B24] Broz AK Broeckling CD De-la-Pena C Lewis MR Greene E Callaway RM Sumner LW Vivanco JM Plant neighbor identity influences plant biochemistry and physiology related to defence BMC Plant Biol 2010 10 115 10.1186/1471-2229-10-115 20565801 PMC3095278

[B25] Ashley MK Grant M Grabov A Plant responses to potassium deficiencies: a role for potassium transport proteins J Exp Bot 2006 57 425 436 16364949 10.1093/jxb/erj034

[B26] Schmelz EA Alborn HT Engelberth J Tumlinson JH Nitrogen deficiency increases volicitin-induced volatile emission, jasmonic acid accumulation, and ethylene sensitivity in maize Plant Physiol 2003 133 295 306 10.1104/pp.103.024174 12970495 PMC196606

[B27] Kieu NP Aznar A Segond D Rigault M Simond-Cote E Kunz C Soulie MC Expert D Dellagi A Iron deficiency affects plant defence responses and confers resistance to Dickeya dadantii and Botrytis cinerea Mol Plant Pathol 2012 in press 10.1111/j.1364-3703.2012.00790.x PMC6638873 22375884

[B28] Rizhsky L Liang H Shuman J Shulaev V Davletova S Mittler R When defence pathways collide. The response of Arabidopsis to a combination of drought and heat stress Plant Physiol 2004 134 1683 1696 10.1104/pp.103.033431 15047901 PMC419842

[B29] Boege K Induced responses to competition and herbivory: natural selection on multi-trait phenotypic plasticity Ecology 2010 91 2628 2637 10.1890/09-0543.1 20957957

[B30] Siemens DH Lischke H Maggiulli N Schürch S Roy BA Cost of resistance and tolerance under competition: the defence-stress benefit hypothesis Evol Ecol 2003 17 247 263 10.1023/A:1025517229934

[B31] Cipollini DF Bergelson J Interspecific competition affects growth and herbivore damage of Brassica napus L. in the field Plant Ecol 2002 162 227 231 10.1023/A:1020377627529

[B32] Agrawal AA Resistance and susceptibility of milkweed: competition, root herbivory, and plant genetic variation Ecology 2004 85 2118 2133 10.1890/03-4084

[B33] Moreno JE Tao Y Chory J Ballaré CL Ecological modulation of plant defence via phytochrome control of jasmonate sensitivity Proc Nat Acad Sci USA 2009 106 4935 4940 10.1073/pnas.0900701106 19251652 PMC2660767

[B34] Coley PD Bryant JP Chapin FS Resource availability and plant antiherbivore defence Science 1985 230 895 899 10.1126/science.230.4728.895 17739203

[B35] Armengaud P Breitling R Amtmann A Coronatine-insensitive 1 (COI1) mediates transcriptional responses of Arabidopsis thaliana to external potassium supply Mol Plant 2010 3 390 405 10.1093/mp/ssq012 20339157 PMC2845782

[B36] Storey JD Tibshirani R Statistical significance for genomewide studies Genome Biol 2003 100 9440 9445 10.1073/pnas.1530509100 PMC170937 12883005

[B37] Du Z Zhou X Lin Y Zhang Z Su Z AgriGO: a GO analysis toolkit for the agricultural community Nucl Acids Res 2010 38 W64 W70 10.1093/nar/gkq310 20435677 PMC2896167

[B38] Liechti R Csárdi G Bergmann S Schütz F Sengstag T Boj SF Servitja J-M Ferrer J Van Lommel L Schuit F EuroDia: a beta-cell gene expression resource Database 2010 2010 baq024 10.1093/database/baq024 20940178 PMC2963318

[B39] Subramanian A Kuehn H Gould J Tamayo P Mesirov JP GSEA-P: a desktop application for Gene Set Enrichment Analysis Bioinformatics 2007 23 3251 3253 10.1093/bioinformatics/btm369 17644558

