# Supplementary figures and images for: Transcriptome analysis of intraspecific competition in Arabidopsis thaliana reveals organ-specific signatures related to nutrient acquisition and general stress response pathways
Source: BMC Plant Biol. 2012 Nov 29;12:227. doi: 10.1186/1471-2229-12-227 (PMC3536592; doi:10.1186/1471-2229-12-227)

a

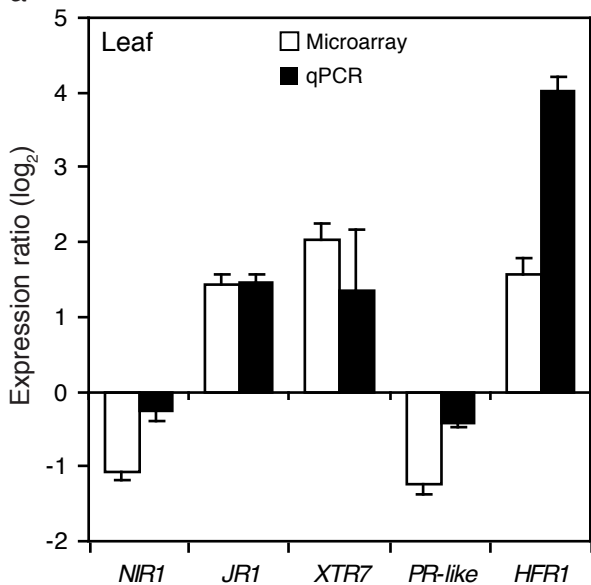

b

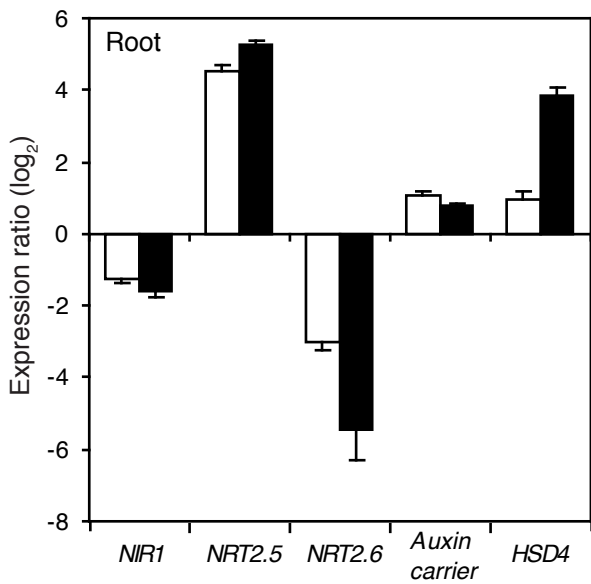

Supplement: Additional file 2 — Validation of microarray data by qPCR. Validation of microarray data by qPCR. Expression of selected genes differentially expressed in leaf and root tissues was analyzed on independent samples by qPCR. Expression ratios are calculated from experiments comparing plants growing at a density of 20 plants per pot and plants growing alone. Values (±SE) are normalized to the reference genes and are the mean of three biological replicates. Microarray data are shown for comparison. [file 1471-2229-12-227-S2.pdf]
