# Supplementary material for: Transcriptome analysis of intraspecific competition in Arabidopsis thaliana reveals organ-specific signatures related to nutrient acquisition and general stress response pathways
Source: BMC Plant Biol. 2012 Nov 29;12:227. doi: 10.1186/1471-2229-12-227 (PMC3536592; doi:10.1186/1471-2229-12-227)
Supplement: Additional file 3 — Gene ontology (GO) analysis of differentially expressed genes. Enrichment of differentially expressed genes in different gene ontology (GO) categories. Genes induced or repressed by competition in both leaf or root samples were classifed in different GO categories with AgriGO singular enrichment analysis. [file 1471-2229-12-227-S3.pdf]

Additional file 2. Enrichment of differentially expressed genes in different gene ontology (GO) categories. Genes induced or repressed by competition in both leaf or root samples were classifed in different GO categories with AgriGO singular enrichment analysis.

GO term	Term	FDR
**Leaf**		
GO:0009628	response to abiotic stimulus	7.90E-15
GO:0042221	response to chemical stimulus	4.70E-13
GO:0050896	response to stimulus	4.30E-12
GO:0006950	response to stress	2.00E-10
GO:0009416	response to light stimulus	4.80E-10
GO:0009314	response to radiation	7.80E-10
GO:0010033	response to organic substance	1.60E-08
GO:0009719	response to endogenous stimulus	1.60E-08
GO:0006979	response to oxidative stress	2.00E-07
GO:0009725	response to hormone stimulus	3.00E-07
GO:0009605	response to external stimulus	8.00E-06
GO:0009791	post-embryonic development	1.30E-05
GO:0010035	response to inorganic substance	2.10E-05
GO:0009639	response to red or far red light	3.50E-05
GO:0000302	response to reactive oxygen species	6.50E-05
GO:0009414	response to water deprivation	7.50E-05
GO:0009642	response to light intensity	8.80E-05
GO:0009415	response to water	1.10E-04
GO:0065008	regulation of biological quality	1.70E-04
GO:0034641	cellular nitrogen compound metabolic process	2.00E-04
**Root**		
GO:0006812	cation transport	2.40E-02
GO:0006811	ion transport	2.40E-02
GO:0006810	transport	2.40E-02
GO:0051234	establishment of localization	2.40E-02
GO:0050896	response to stimulus	2.40E-02
GO:0006950	response to stress	2.40E-02
GO:0051179	localization	2.40E-02
GO:0006790	sulfur metabolic process	2.40E-02
GO:0010035	response to inorganic substance	4.70E-02
GO:0042221	response to chemical stimulus	5.20E-02
GO:0051707	response to other organism	5.40E-02
GO:0009607	response to biotic stimulus	8.30E-02
GO:0006979	response to oxidative stress	9.10E-02
GO:0019748	secondary metabolic process	9.10E-02
GO:0009611	response to wounding	9.10E-02
GO:0010033	response to organic substance	1.00E-01
GO:0009605	response to external stimulus	1.00E-01
GO:0009753	response to jasmonic acid stimulus	1.20E-01
GO:0030001	metal ion transport	1.30E-01
GO:0010038	response to metal ion	1.60E-01
