# Supplementary material for: Transcriptome analysis of intraspecific competition in Arabidopsis thaliana reveals organ-specific signatures related to nutrient acquisition and general stress response pathways
Source: BMC Plant Biol. 2012 Nov 29;12:227. doi: 10.1186/1471-2229-12-227 (PMC3536592; doi:10.1186/1471-2229-12-227)
Supplement: Additional file 5 — Lamp specifications. Spectral composition of the Osram Lumilux L58W/830 Warm White lamps. [file 1471-2229-12-227-S5.pdf]

Osram Lumilux L58W/830 Warm White lamps

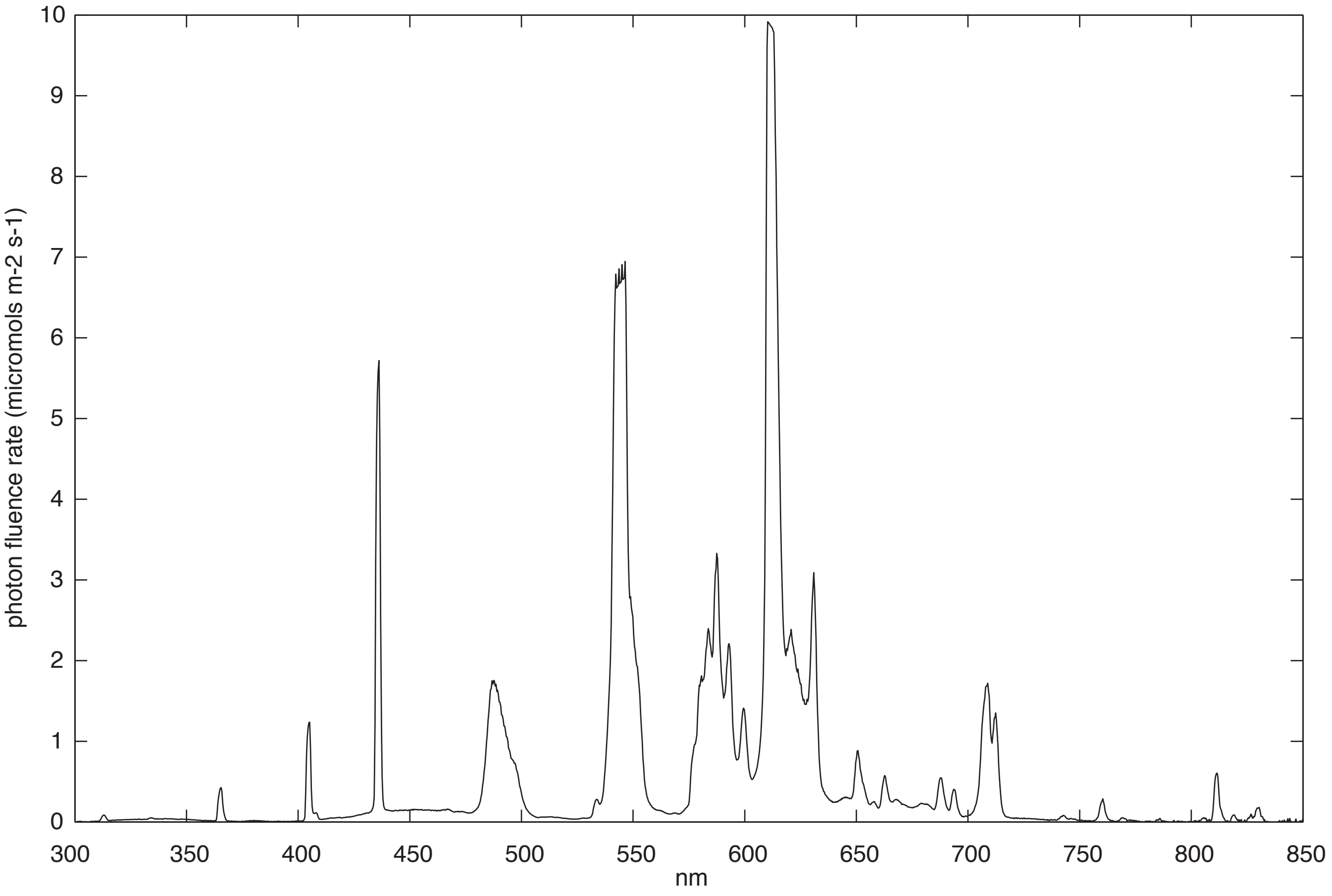

The spectral light composition in the plant growth room was measured using a USB2000+ UV-VIS spectrometer (Ocean Optics, Inc., Dunedin, USA). For each measurement, 50 spectra were recorded at plant level and averaged to a mean spectrum.

R radiation was computed from 600nm to 680nm and FR from 680nm to 760nm.

R/FR ratio = 6.9
