# Supplementary material for: Transcriptome analysis of intraspecific competition in Arabidopsis thaliana reveals organ-specific signatures related to nutrient acquisition and general stress response pathways
Source: BMC Plant Biol. 2012 Nov 29;12:227. doi: 10.1186/1471-2229-12-227 (PMC3536592; doi:10.1186/1471-2229-12-227)
Supplement: Additional file 6 — List of primers used for qPCR analysis. [file 1471-2229-12-227-S6.pdf]

Additional file 5. List of primers used for QPCR analyses

Gene	AGI	Orientation	Sequence (5' - 3')
NIR1	At2g15620	foward	CATGGGATGCTTAACACGAG
		reverse	ATCTCAGCCACCAATGGAAC
JR1	At3g16470	foward	AGATGGGATTCTGGTCGTTG
		reverse	ACGATCAGAGGCCATGAAAG
XRT7	At4g14130	foward	ATCGTGGCGACTGTTCTTCT
		reverse	CCAGCGACAAAGACAGCATA
PR-like	At1g20030	foward	TCTGTCCTTCTCCCAACACC
		reverse	AAGCAAGCGAGAGAAGAACG
HFR1	At1g02340	foward	ACAAGACGGACAAGGTTTCG
		reverse	GTCGCCGGAAGAAAATAAGG
NRT2.5	At1g12940	foward	CTTTCGCCGTTCTCTTGTTC
		reverse	TCCCCACATCATCTTTCTCC
NRT2.6	At3g45060	foward	ACCTGACCAAAACCGACATC
		reverse	TGAGAAGGCGGTTCCATAAC
Auxin carrier	At2g17500	foward	AGATGGGATTCTGGTCGTTG
		reverse	ACGATCAGAGGCCATGAAAG
HSD4	At5g50590	foward	ACTTTTGGGGACCGGTTTAC
		reverse	CATAGCTGCTTTGCTTGCAG
GAPDH	At1g13440	foward	TTGGTGACAACAGGTCAAGCA
		reverse	AAACTTGTCGCTCAATGCAATC
SAND	At2g28390	foward	AACTCTATGCAGCATTTGATCCACT
		reverse	TGATTGCATATCTTTATCGCCATC
